# Cytotoxic Glucose Degradation Products in Fluids for Peritoneal Dialysis

**Published:** 2011

**Authors:** Noushin Adib, Maryam Shekarchi, Homa Hajimehdipoor, Gloria Shalviri, Maral Shekarchi, Maryam Imaninejad

**Affiliations:** a *Food and Drug Lab Research Center, Ministry of Health, Tehran, Iran.*; b *Adverse Drug Reaction Center, Food and Drug Deputy, Ministry of Health, Tehran, Iran.*

**Keywords:** Chemical peritonitis, Glucose degradation products, Peritoneal dialysis, Acetaldehyde

## Abstract

During the standard heat sterilization process of the lactate–buffered peritoneal dialysis solutions, glucose (an osmotic active substance) degrades to form compounds called glucose degradation products which are cytotoxic and affect the survival of the peritoneal membrane. This case presentation is based on an observation of 224 aseptic peritonitis cases of unknown etiology. For the purpose of clarification, we analyzed the peritoneal dialysis solutions for the presence of acetaldehyde by using a developed and validated high-performance liquid chromatography (HPLC) pre-column derivitazation. The method was validated with respect to validation factors such as linearity, precision, recovery and (LOD). The acetaldehyde level of solutions before heat sterilization was 1.78 ± 2.7 ppm whereas in samples after heat sterilization was about 20 ± 2.07 ppm. Based on the forementioned findings, we hypothesized that the higher levels of acetaldehyde and possibly the other glucose degradation products may have been an etiological factor in these 224 cases of chemical peritonitis. So it is important for the manufacturers to carefully review the heat of sterilization process in the production line.

## Introduction

Peritoneal dialysis is prescribed for patients with acute or chronic renal failure when nondialytic medical therapy is judged to be inadequate (1). It may also be used in the treatment of certain fluid and electrolyte disturbances, and for the patients intoxicated with certain poisons and drugs (2). During chronic peritoneal dialysis (PD), the peritoneum is continuously exposed to various insults mostly rooted in non-physiological nature of the PD solutions. Conventional PD fluids (PDFs) contain glucose as the osmotic agent and lactate as the buffer base (3). Besides the high osmolarity and the low pH of the dialysis solution, unusual heat sterilization glucose degradation products GDPs such as formaldehyde, acetaldehyde, 5-hydroxymethyl-2-furfural, glyoxal, 2-furaldehyde, methylglyoxal, and some still-unidentified biologically active compounds generated from glucose are considered to be harmful for the peritoneal membrane (4-12). The GDPs interact with protein giving rise to the formation of so-called advanced glycation end products (AGEs). The accumulation of AGEs may result in the progression of peritoneal interstitial fibrosis and vascular sclerosis (13). This breakdown process is also called caramelization and may give the fluid a caramel-like, darker, yellowish brown color (13). This observation was carried out after the complaint of 224 renal failure patients (104 females and 120 males) who have been under continuous (PD) treatment. The patients showed the symptoms of peritonitis, including cloudy dialyzate effluents (224 cases), abdominal discomfort with additional pain (204 cases), vomiting and temporary loss of ultra filtrate, and dialysis cell counts of 100-2800 white blood cell per micro liter. The sterility test of the product and the gram strains from all patients revealed no microorganisms. No associated rash, fever or other hypersensitivity reactions were present. Antibiotic therapy was performed for patients developed peritonitis following initial symptoms of the reaction (mostly ceftazidim, cefazolin or amikacin), however the challenge of receiving the solution was necessary for all patients. It was remarkable that peritonitis signs were evident only in patients using bags with one special lot number so we suspected an aseptic peritonitis induced by one or more chemicals in PD fluids. The PD bag manufacturer was immediately informed of the problem and fluid samples were taken. The glucose degradation, pH and the acetaldehyde levels were investigated in the PD fluids of two lots before and after sterilization. Low-molecular-weight aldehydes such as formaldehyde and acetaldehyde are extremely difficult to extract from an aqueous solution with an organic solvent as they are soluble both in water and in lipids. Therefore, it is necessary to prepare more stable and less reactive derivatives. The most common reagents for derivitazation, 2, 4-dinitrophenylhydrazine (14), 2-mino ethanthiol (cysteamine) (15, 16) and *o*-(2, 3, 4, 5, 6-pentafluorobenzyl)-hydroxylamine (17, 18) form hydrazones, thiazolines and hydroximes, respectively after reacting with aldehyde. There have been numerous articles on the use of 2, 4-dinitrophenylhydrazine derivatives of reactive carbonyl compounds in various samples, including water, air, foods, tissues and blood (19-21). So in this study a pre-column derivitazation (HPLC) method has been developed and validated for determination of acetaldehyde in three different batches of complained PD-fluids, before and after heat sterilization.

## Experimental

*Reagents*

Acetonitrile, methanol, *n*-pentane (HPLC grade) and hydrochloric acid were purchased from Merck Co. (Merck, Germany). Acetaldehyde and 2,4-dinitrophenylhydrazine were purchased from Aldrich chemical. The samples were collected from internal manufacturer who was responsible for this case report (three batches).

*Instrument*

HPLC experiments were performed using a Water Alliance system equipped with a vacuum degasser, a quaternary solvent mixing, an auto sampler and a waters 2996 diode array detector. The ultraviolet (UV) spectra were collected across the range of 200-900 nm, extracting 330 nm for chromatograms. Empower software was utilized for instrument control, data collection and data process. The column was a μ-Bondapak C_18 _(10 μm particle size, 12.5 cm × 4.6 mm I.D.). The elution of the compounds was performed at a constant flow rate of 1 mL/min by using acetonitrile : water (49 : 51) v/v mobile phase**. **

*Hydrazones standards*

Synthetic hydrazones derivatives were prepared (19) by reacting of 30 mL of 2, 4-dinitrophenylhydrazine (DNPH) stock solution with an excess amount of acetaldehyde. The reaction proceeded rapidly at room temperature. The precipitated hydrazones were filtered, dried and crystalized from methanol-water. A solution of 100 μg/mL of hydrazone was prepared and chromatographed as described. 

*Sample preparation*

For derivatization, 310 mg of 2, 4-dinitrophenylhydrazine was dissolved in 100 mL of 2 M HCL, and 0.2 mL of this reagent was added to 1 mL of peritoneal dialysis fluid, 8.8 mL water and 20 mL of *n*-pentane in 50 mL screw-capped PTFE-lined tubes (polytetra fluoro ethylene). The tubes were shaken for 30 min, and the organic layers were removed. The aqueous layers were extracted with additional 20 mL of *n-*pentane. The *n-*pentane extracts were combined, evaporated under vacuum and reconstituted in 0.4 mL of acetonitrile. A 20 μL of each samples injected to the HPLC column and washed isocratically with eluent as described above.

*Validation*

The reliability of HPLC-method was validated through its linearity, sensitivity, precision and recovery.

*Linearity*

Due to the verification of the normal distribution of the results, linearity was evaluated through the relationship between the concentration of ascorbic acid and the absorbance obtained from the UV-HPLC detector. The determination coefficient (r^2^) was calculated by means of the least- squares analysis. The calibration line was done through two replicates of each concentration of acetaldehyde hydrazone (20-110 ppm) to know the extent of the total variability of the response that could be explained by the linear regression model.

*Sensitivity*

The detection limit and the quantification limit were calculated from the calibration curve that defined linearity.

*Precision*

The precision of the method indicates the amount of dispersion within a series of determinations of the same sample. Six samples were analyzed in the same day and the relative standard deviations (RSD) were calculated dividing the standard deviation by the mean of the concentration.

*Recovery*

This parameter showed the proximity between the experimental values and the real ones. It ensured that no loss or uptake occurred during the process. The determination of this parameter was performed for the method through studying the recovery after a standard addition procedure with two addition levels. The concentrations of acetaldehyde hydrazone added to the samples were 20 and 50 ppm in each addition level, six determinations were carried out and the recovery percent was calculated in each case.

*Statistical analysis*

Data have been reported as mean ± SD. The results were analyzed statistically by SPSS software and the Student t-test with level of significance set at p < 0.05.

## Results and Discussion

**Figure 1:**
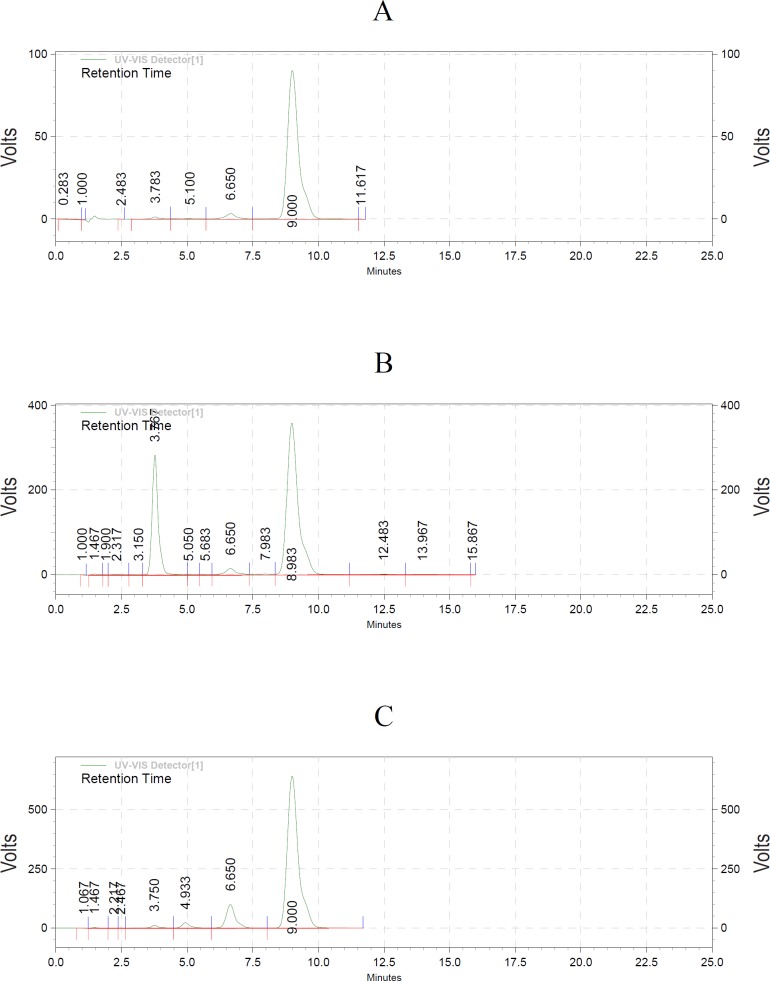
(A) The standard of acetaldehyde hydrazone (9 min). (B) Spiking 2,4-dinitrophenylhydrazine (3.7 min) and acetaldehyde hydrazon (8.9 min). (C) Peritoneal dialysis sample.

Utilizing HPLC elution method, the acetaldehyde hydrazone was separated and registered by photodiode array detector (Figure 1). The extraction recoveries of acetaldehyde hydrazone were 95% and 98% respectively. Since most of the compound produced in PD-fluid was acetaldehyde, the calibration curve for acetaldehyde was generated. Each dilution was injected and chromatographed in triplicate. The concentration of each standard was plotted against the peak areas. The calibration graph was linear with r^2^ = 0.9993. The low RSD% (1.8) obtained from experiments showed precision of the analytical measurement. The amount of acetaldehyde in three different lots PD-solutions before and after sterilization is indicated in Table1. Statistical analysis demonstrated significant differences between acetaldehyde content in peritoneal solutions before and after sterilization (p < 0.05).

Following feedback from iranian adverse drug reaction center (ADR), about 224 cases of chemical peritonitis, procedural changes were made in the newly established production line and no similar problems have occurred since. Although it is not clear which particular glucose degradation product is responsible for their cytotoxic effects (7), it is well known that the glucose degradation products in commercially available PD-solutions may contribute to peritoneal membrane injury (22). There is only limited clinical evidence to prove that any GDPs have induced PD-fluid side-effects in human beings, including reversible loss of ultra filtration and abdominal pain induced by PD-bags towards the end of their shelf life (23, 24). There was only one case report about GDPs, in concentration 3-4 times higher than the usual in commercial PD-bags, induced acute chemical peritonitis (13)**. **

**Table 1:** Amount of acetaldehyde in three different Lots of peritoneal dialysis solutions,* n = 3

	**Before sterilization** ^*^	**After sterilization** ^*^
Lot 1	1.76±1.1	19.9±1.5
Lot 2	1.69±1.5	19.7±1.3
Lot 3	1.78±1.8	20.5±1.7

Our findings and observations confirm that the acetaldehyde concentration about 10 times higher than usual may have been the reason of chemical peritonitis in complained patients. In order to avoid patient complains, the manufacturer reviewed the heat of sterilization process in the production line. Following the revision in the heat sterilization acetaldehyde concentration in bags below 5 ppm, no further unusual patient complaints occurred during the use of locally produced PD-fluids in Iran. It is proved that the commercially available single-chamber bag peritoneal dialysis fluids have cytotoxic effects in *in-vitro *studies because of the electrolytes and glucose as osmotic agents, which convert to glucose degradation products during heat sterilization and storage (3, 25). The main strategies to improve biocompatibility have been reduction or elimination of glucose degradation products. The acetaldehyde formation can be prevented by a separation of the reaction partners, glucose and lactate, in double-chamber bags. In double-chamber bags a neutral physiological pH can be achieved after mixing the compartments. Therefore, it is suggested that the responsible company should use double-chamber modern technology, or improve the heat-sterilization process.

## References

[1] Vaamonde CA, Perez GO (1977). Peritoneal dialysis today. Kidney.

[2] Winchester JF, Gelfand MC, Knepshield JH, Schreiner GE (1977). Dialysis and hemoperfusion of poisons and drugs-update. Trans. Am. Soc. Artif. Intern. Organs.

[3] Crawford-Bonadio TL, Diaz-Buxo JA (2004). Comparison of peritoneal dialysis solutions. Nephrol. Nurs. J.

[4] Kjellstrand P, Erixon M, Wieslander A, Linden T, Martinson E (2004). Temperature: the single most important factor for degradation of glucose fluids during storage. Perit. Dial. Int.

[5] Griffin JC, Marie SC (1958). Glucose degradation in the presence of sodium lactate during autoclaving at 121°C. Am. J. Hosp. Pharm.

[6] Wieslander A, Forsback G, Svenson E, Linden T (1996). Cytotoxicity, pH, and glucose degradation products in four different brands of PD fluid. Adv. Perit. Dial.

[7] Nafissi Varcheha N, Shafaatib A, Zarghib A, Aboofazelia R (2004). Separation of somatropin and its degradation products by high-performance liquid chromatography using a reversed-phase polymeric column. Iranian J. Pharm. Res.

[8] Kjellstrand P, Martinson E, Wieslander A, Holmquist B (1995). Development of toxic degradation products during heat sterilization of glucose-containing fluids for peritoneal dialysis: influence of time and temperature. Perit. Dial. Int.

[9] Simonsen O, Sterner G, Carlsson O, Wieslander A, Rippe B (2006). Improvement of peritoneal ultrafiltration (UF) with a PD-solution buffered with a Bicarbonate (B)/Lactate (L) mixture. Perit. Dial. Int.

[10] Erixon M, Wieslander A, Linden T, Carlsson O, Forsback G, Svensson E, Jonsson JA, Kjellstrand P (2005). Take care in how you store your PD fluids: actual temperature determines the balance between reactive and non-reactive GDPs. Perit. Dial. Int.

[11] Erixon M, Linden T, Kjellstrand P, Carlsson O, Ernebrant M, Forsback G, Wieslander A, Jonsson JA (2004). PD fluids contain high concentrations of cytotoxic GDPs directly after sterilization. Perit. Dial. Int.

[12] Sitter T, Sauter M (2005). Impact of glucose in peritoneal dialysis: saint or sinner?. Perit. Dial. Int.

[13] Tuncer M, Sarikaya M, Sezer T, Ozcan S, Suleymanlar G, Yakupoglu G (2000). Chemical peritonitis associated with high dialysate acetaldehyde concentrations. Nephrol. Dial. Transplant.

[14] Kuwata K, Uebori M, Yamasaki H, Kuge Y (1983). Determination of aliphatic aldehydes in air by liquid chromatography. Anal. Chem.

[15] Hayashi T, Reece C, Shibamoto T (1986). Gas chromatographic determination of formaldehyde in coffee via thiazolidine derivative. J. Assoc. Off. Anal. Chem.

[16] Yasuhara A, Shibamoto T (1989). formaldehyde quantization in air samples by thiazoline derivitazation. J. Assoc. Off. Anal. Chem.

[17] Kobayashi K, Tanaka M, Kawai S (1980). Gas chromatographic determination of low molecular weight carbonyl compounds in aqueous solution as their o-(2,3,4,5,6-pentafluorobenzyl) oximes. J. Chromatogr.

[18] Nishikawa H, Yasuhara A (1995). Derivitazation and gas chromatographic or high performance liquid chromatographic analysis of aldehydes in air samles. J. Environ. Chem.

[19] Nilsson-Thorell C, Muscalu N, Andren AH, Kjellstrand PT, Wieslander AP (1993). Heat sterilization of fluids for peritoneal dialysis gives rise to aldehydes. Perit. Dial. Int.

[20] Shibamoto T (2006). Analytical methods for trace levels of reactive carbonyl compounds formed in lipid peroxidation systems. J. Pharm. Biomed. Anal.

[21] Vogel M, Buldt A, Karst U (2000). Hydrazine reagents as derivatizing agents in environmental analysis a critical review. Fresenius J. Anal. Chem.

[22] Wieslander AP, Nordin MK, Kjellstrand PT, Boberg UC (1993). Heat sterilized PD-fluids impair growth and inflammatory responses of cultured cell lines and human leukocytes. Clin. Nephrol.

[23] Henderson IS, Couper IA, Lumsden A (1985). Frontiers in Peritoneal Dialysis. Field, Rich, New York.

[24] Henderson IS, Couper IA, Lumsden A, La Greca G (1986). The effect of shelf life of peritoneal dialysis fluid on ultra-filtration in CAPD. Peritoneal Dialysis.

[25] Cooker LA, Luneburg P, Faict D, Choo C, Holmes CJ (1997). Reduced glucose degradation products in bicarbonate/lactate-buffered peritoneal dialysis solutions produced in two- chambered bags. Perit. Dial. Int.

